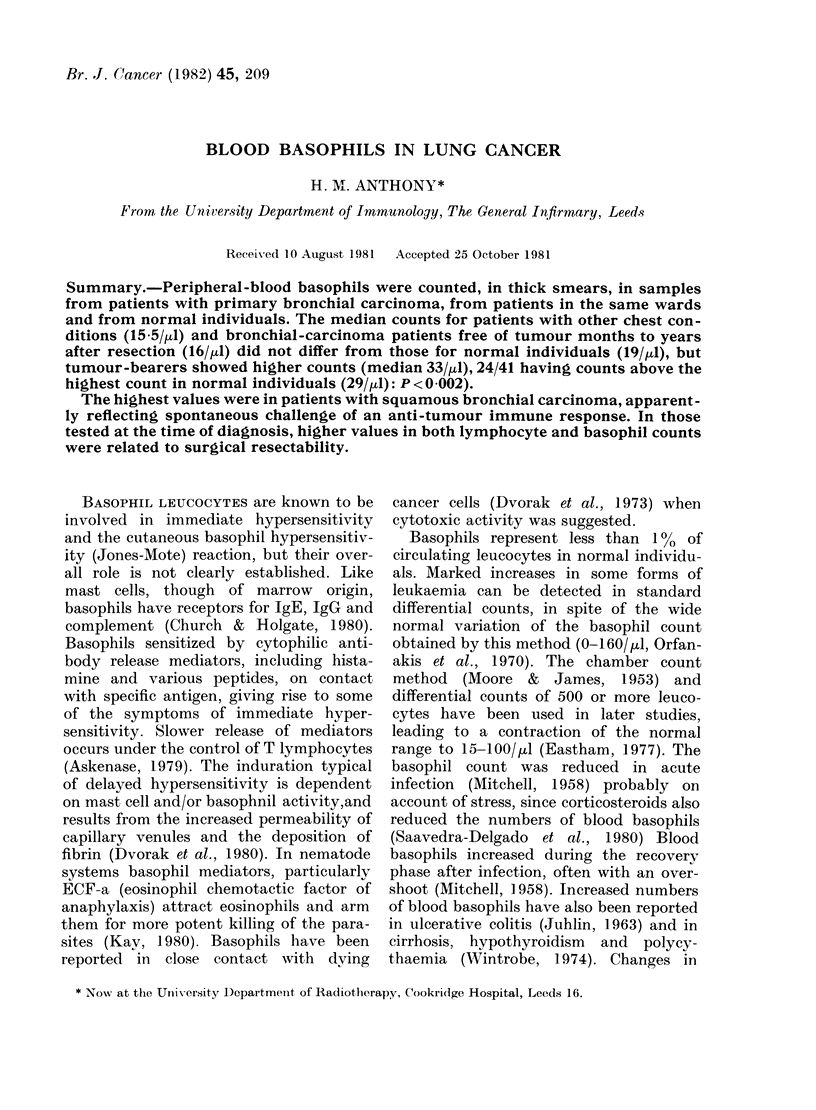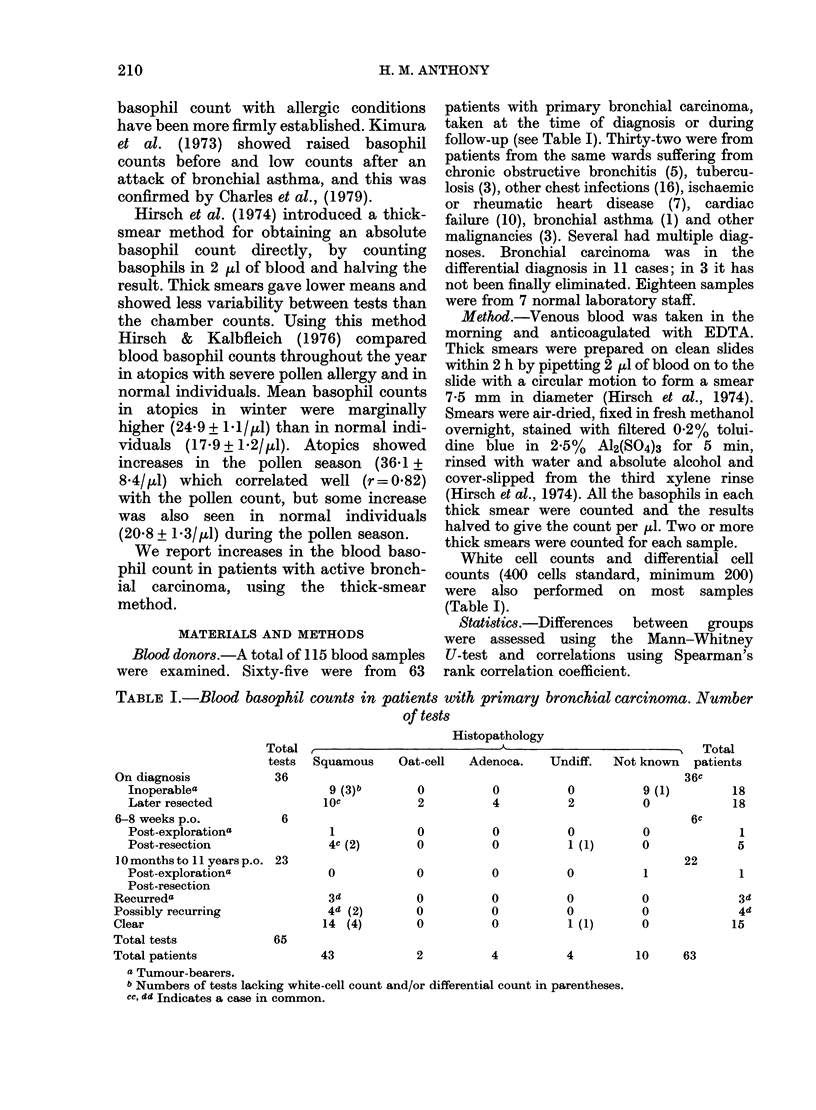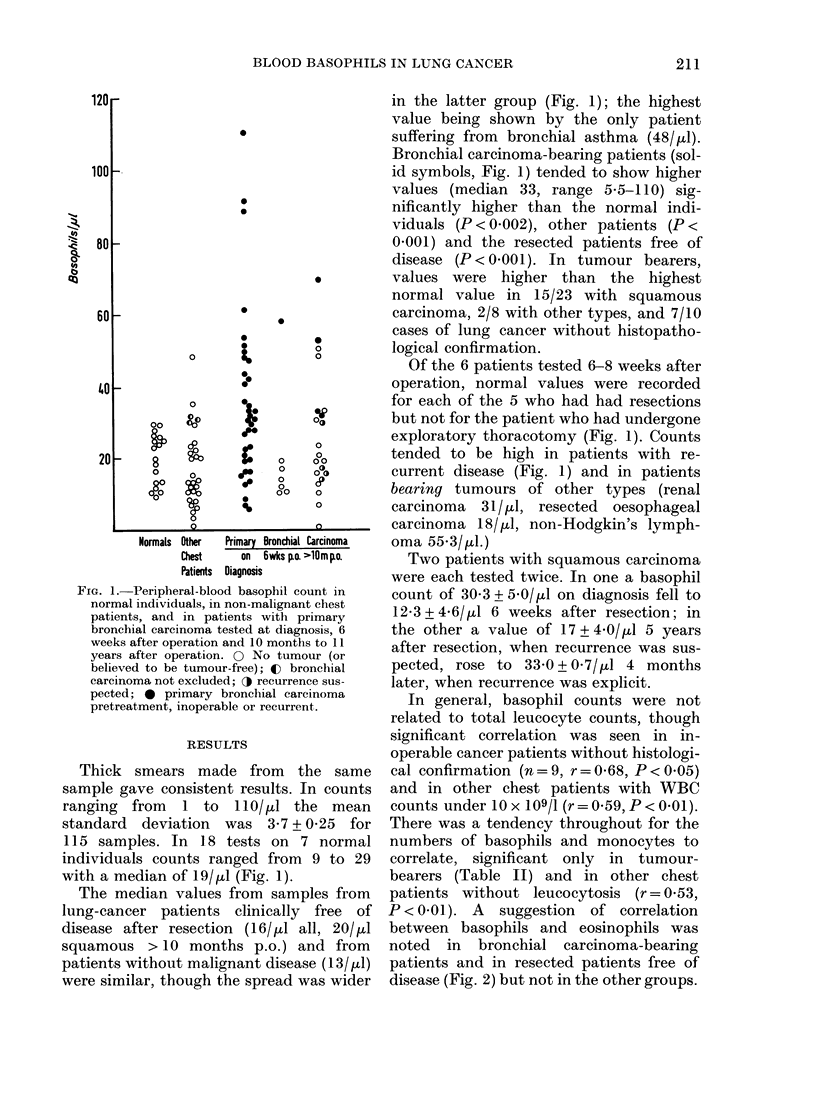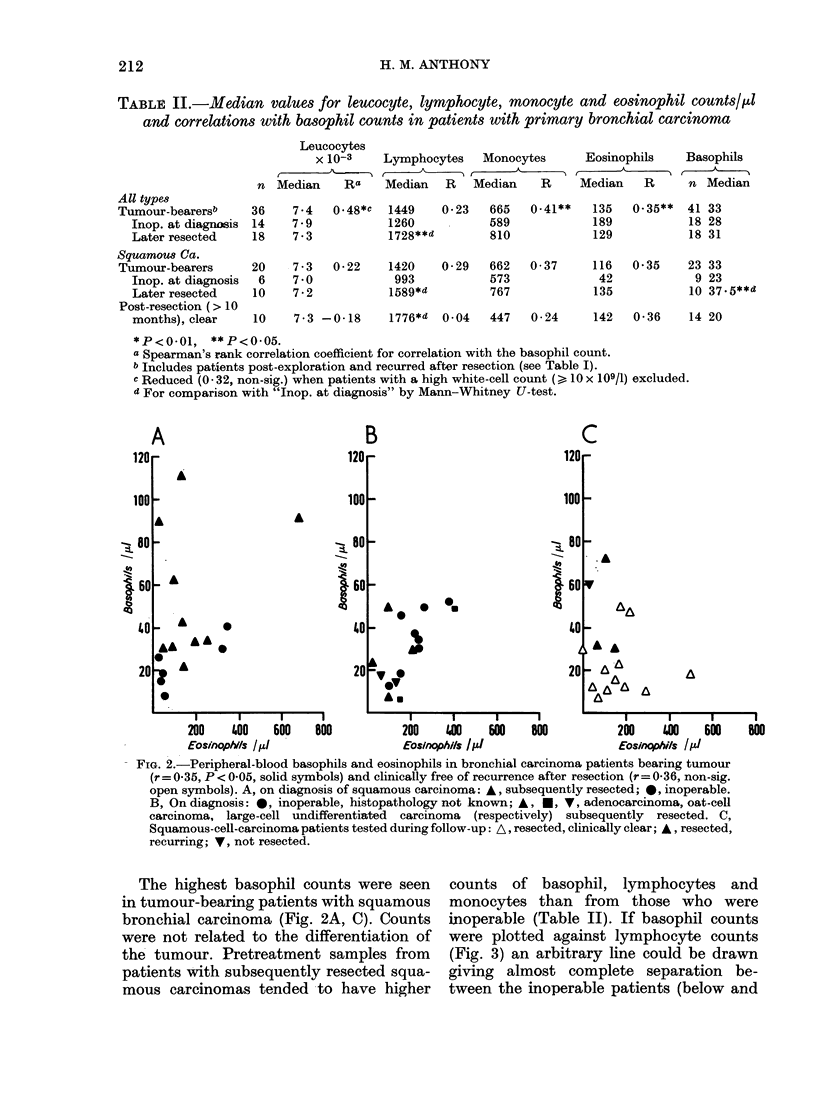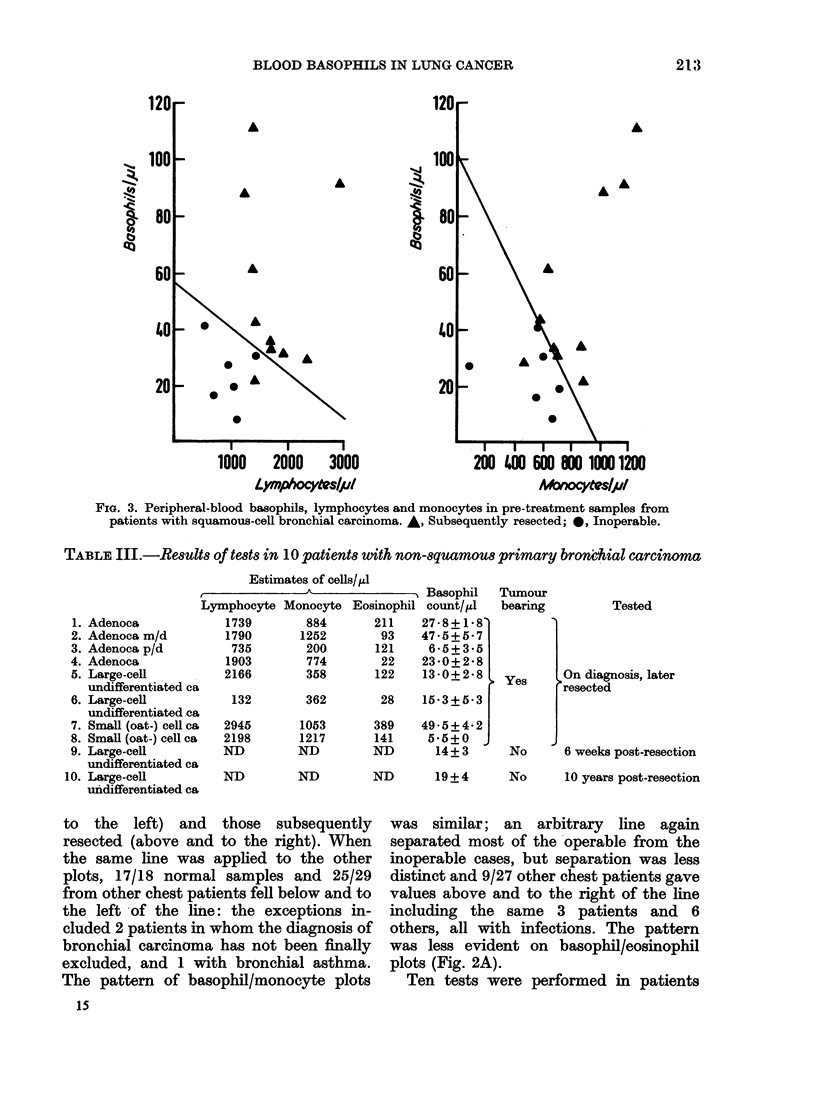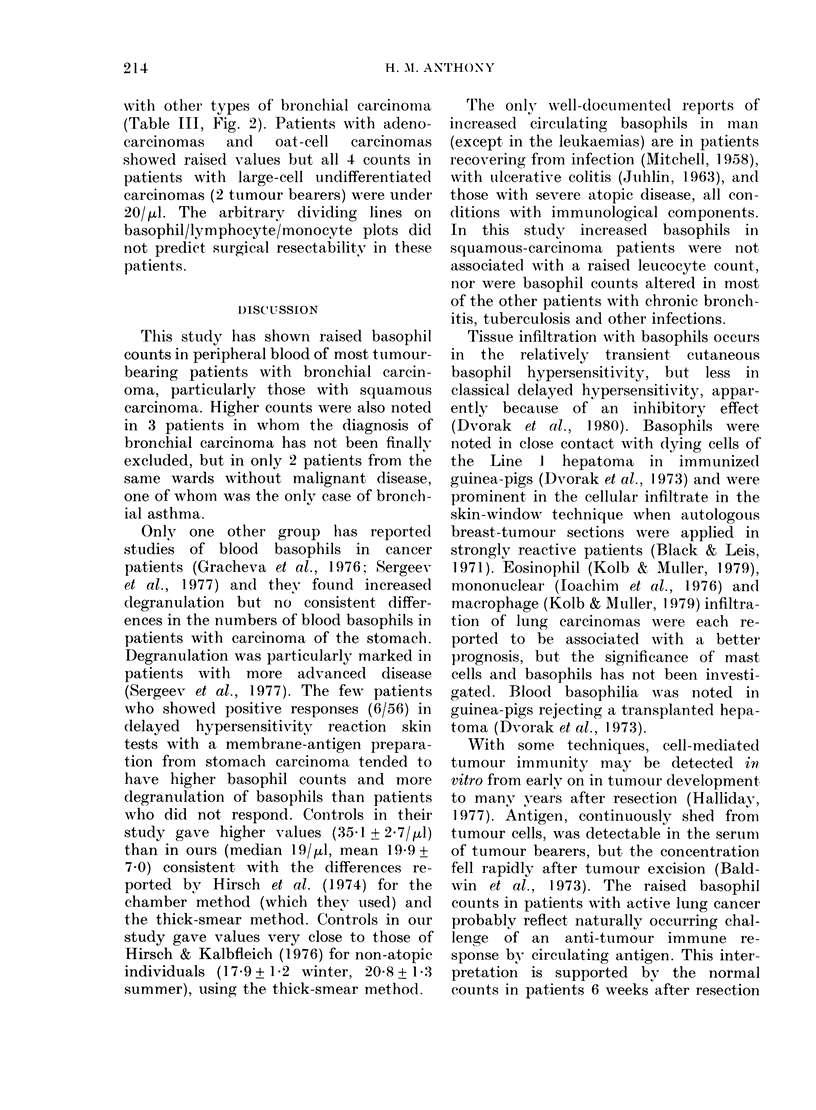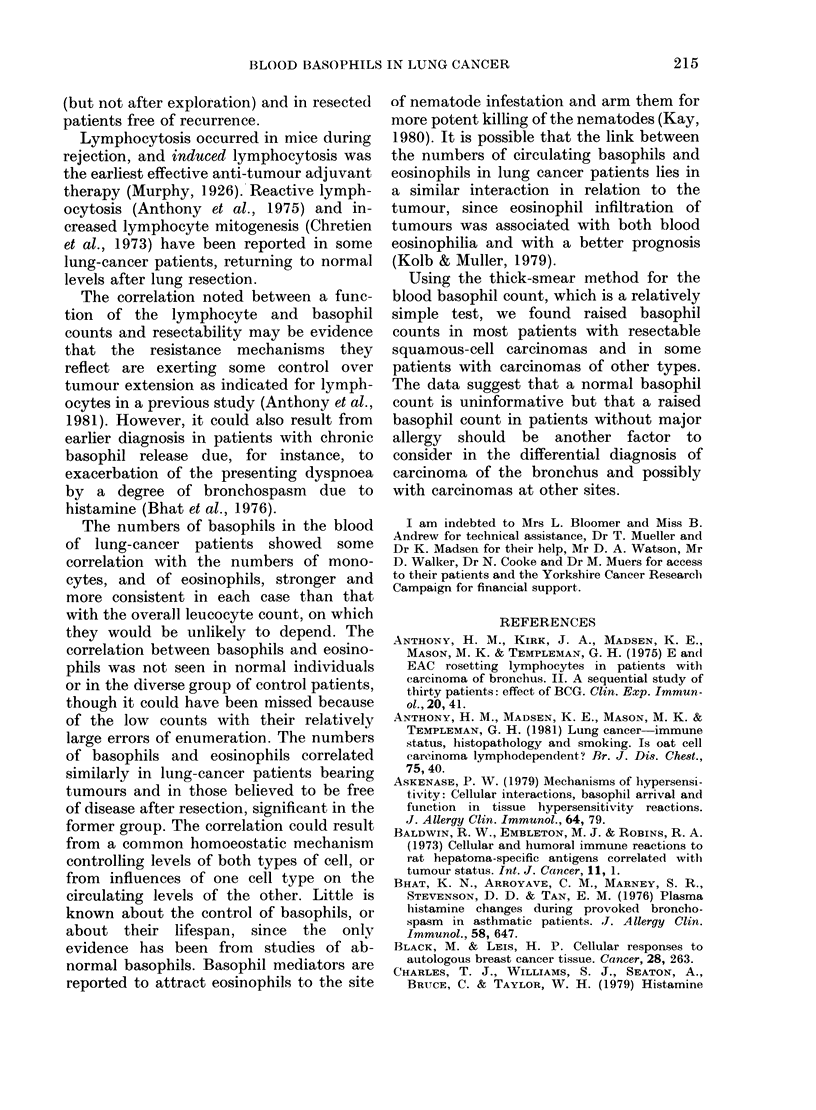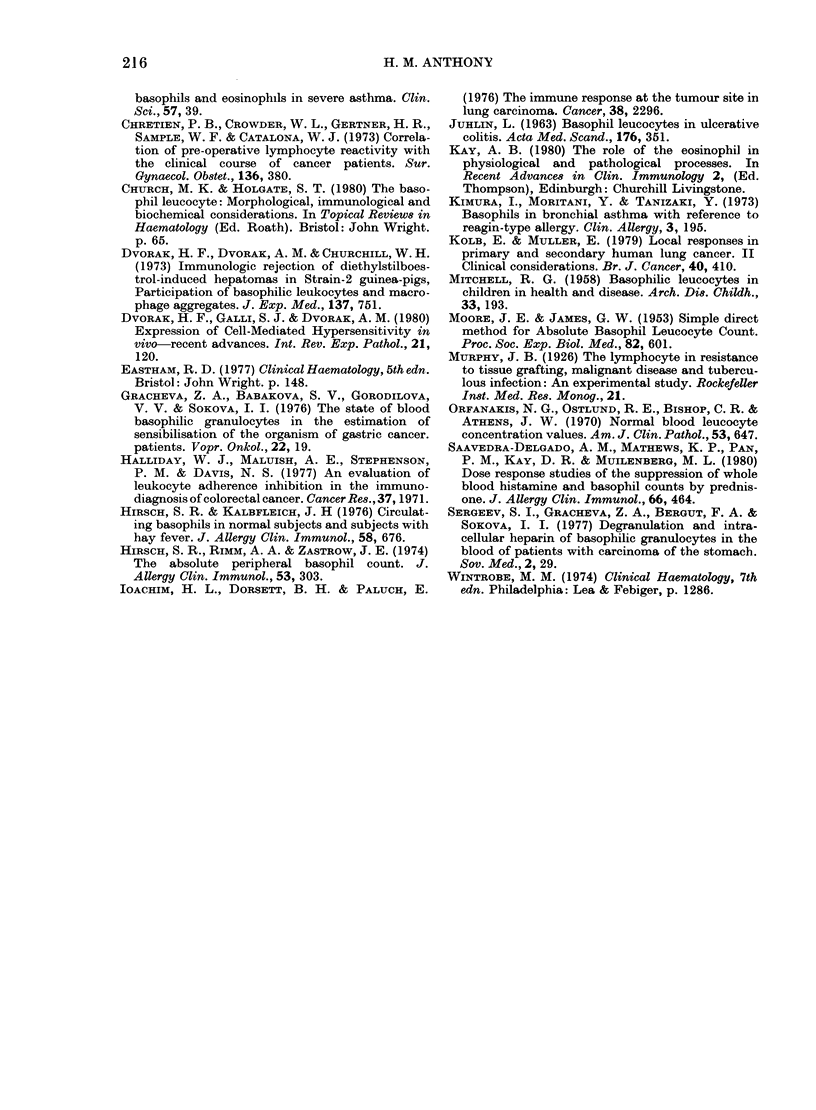# Blood basophils in lung cancer.

**DOI:** 10.1038/bjc.1982.35

**Published:** 1982-02

**Authors:** H. M. Anthony

## Abstract

Peripheral-blood basophils were counted, in thick smears, in samples from patients with primary bronchial carcinoma, from patients in the same wards and from normal individuals. The median counts for patients with other chest conditions (15.5/microliter) and bronchial-carcinoma patients free of tumour months to years after resection (16/microliter) did not differ from those for normal individuals (19/microliter), but tumour-bearers showed higher counts (median 33/microliter), 24/41 having counts above the highest count in normal individuals (29/microliter): P less than 0.002). The highest values were in patients with squamous bronchial carcinoma, apparently reflecting spontaneous challenge of an anti-tumour immune response. In those tested at the time of diagnosis, higher values in both lymphocyte and basophil counts were related to surgical resectability.


*Br. J. Cancer* (1982) **45**, 209

### BLOOD BASOPHILS IN LUNG CANCER

H. M. ANTHONY*

*From the University Department of Immunology, The General Infirmary, Leeds*

Received 10 August 1981 Accepted 25 October 1981

**Summary.—Peripheral-blood basophils were counted, in thick smears, in samples from patients with primary bronchial carcinoma, from patients in the same wards and from normal individuals. The median counts for patients with other chest conditions (15·5/μl) and bronchial-carcinoma patients free of tumour months to years after resection (16/μl) did not differ from those for normal individuals (19/μl), but tumour-bearers showed higher counts (median 33/μl), 24/41 having counts above the highest count in normal individuals (29/μl): $P < 0 \cdot 002$).**

**The highest values were in patients with squamous bronchial carcinoma, apparently reflecting spontaneous challenge of an anti-tumour immune response. In those tested at the time of diagnosis, higher values in both lymphocyte and basophil counts were related to surgical resectability.**

BASOPHIL LEUCOCYTES are known to be involved in immediate hypersensitivity and the cutaneous basophil hypersensitivity (Jones-Mote) reaction, but their overall role is not clearly established. Like mast cells, though of marrow origin, basophils have receptors for IgE, IgG and complement (Church & Holgate, 1980). Basophils sensitized by cytophilic antibody release mediators, including histamine and various peptides, on contact with specific antigen, giving rise to some of the symptoms of immediate hypersensitivity. Slower release of mediators occurs under the control of T lymphocytes (Askenase, 1979). The induration typical of delayed hypersensitivity is dependent on mast cell and/or basophnil activity,and results from the increased permeability of capillary venules and the deposition of fibrin (Dvorak *et al.*, 1980). In nematode systems basophil mediators, particularly ECF-a (eosinophil chemotactic factor of anaphylaxis) attract eosinophils and arm them for more potent killing of the parasites (Kay, 1980). Basophils have been reported in close contact with dying cancer cells (Dvorak *et al.*, 1973) when cytotoxic activity was suggested.

Basophils represent less than 1% of circulating leucocytes in normal individuals. Marked increases in some forms of leukaemia can be detected in standard differential counts, in spite of the wide normal variation of the basophil count obtained by this method (0–160/μl, Orfanakis *et al.*, 1970). The chamber count method (Moore & James, 1953) and differential counts of 500 or more leucocytes have been used in later studies, leading to a contraction of the normal range to 15–100/μl (Eastham, 1977). The basophil count was reduced in acute infection (Mitchell, 1958) probably on account of stress, since corticosteroids also reduced the numbers of blood basophils (Saavedra-Delgado *et al.*, 1980) Blood basophils increased during the recovery phase after infection, often with an overshoot (Mitchell, 1958). Increased numbers of blood basophils have also been reported in ulcerative colitis (Juhlin, 1963) and in cirrhosis, hypothyroidism and polycythaemia (Wintrobe, 1974). Changes in

* Now at the University Department of Radiotherapy, Cookridge Hospital, Leeds 16.

basophil count with allergic conditions have been more firmly established. Kimura *et al.* (1973) showed raised basophil counts before and low counts after an attack of bronchial asthma, and this was confirmed by Charles *et al.*, (1979).

Hirsch *et al.* (1974) introduced a thick-smear method for obtaining an absolute basophil count directly, by counting basophils in 2 $\mu$l of blood and halving the result. Thick smears gave lower means and showed less variability between tests than the chamber counts. Using this method Hirsch & Kalbfleich (1976) compared blood basophil counts throughout the year in atopics with severe pollen allergy and in normal individuals. Mean basophil counts in atopics in winter were marginally higher ($24 \cdot 9 \pm 1 \cdot 1/\mu$l) than in normal individuals ($17 \cdot 9 \pm 1 \cdot 2/\mu$l). Atopics showed increases in the pollen season ($36 \cdot 1 \pm 8 \cdot 4/\mu$l) which correlated well ($r = 0 \cdot 82$) with the pollen count, but some increase was also seen in normal individuals ($20 \cdot 8 \pm 1 \cdot 3/\mu$l) during the pollen season.

We report increases in the blood basophil count in patients with active bronchial carcinoma, using the thick-smear method.

### MATERIALS AND METHODS

*Blood donors.*—A total of 115 blood samples were examined. Sixty-five were from 63 patients with primary bronchial carcinoma, taken at the time of diagnosis or during follow-up (see Table I). Thirty-two were from patients from the same wards suffering from chronic obstructive bronchitis (5), tuberculosis (3), other chest infections (16), ischaemic or rheumatic heart disease (7), cardiac failure (10), bronchial asthma (1) and other malignancies (3). Several had multiple diagnoses. Bronchial carcinoma was in the differential diagnosis in 11 cases; in 3 it has not been finally eliminated. Eighteen samples were from 7 normal laboratory staff.

*Method.*—Venous blood was taken in the morning and anticoagulated with EDTA. Thick smears were prepared on clean slides within 2 h by pipetting 2 $\mu$l of blood on to the slide with a circular motion to form a smear 7·5 mm in diameter (Hirsch *et al.*, 1974). Smears were air-dried, fixed in fresh methanol overnight, stained with filtered 0·2% toluidine blue in 2·5% $Al_2(SO_4)_3$ for 5 min, rinsed with water and absolute alcohol and cover-slipped from the third xylene rinse (Hirsch *et al.*, 1974). All the basophils in each thick smear were counted and the results halved to give the count per $\mu$l. Two or more thick smears were counted for each sample.

White cell counts and differential cell counts (400 cells standard, minimum 200) were also performed on most samples (Table I).

*Statistics.*—Differences between groups were assessed using the Mann–Whitney $U$-test and correlations using Spearman's rank correlation coefficient.

TABLE I.—*Blood basophil counts in patients with primary bronchial carcinoma. Number of tests*

| | Total tests | Histopathology: Squamous | Oat-cell | Adenoca. | Undiff. | Not known | Total patients |
|---|---|---|---|---|---|---|---|
| On diagnosis | 36 | | | | | | 36[c] |
| Inoperable[a] | | 9 (3)[b] | 0 | 0 | 0 | 9 (1) | 18 |
| Later resected | | 10[c] | 2 | 4 | 2 | 0 | 18 |
| 6–8 weeks p.o. | 6 | | | | | | 6[c] |
| Post-exploration[a] | | 1 | 0 | 0 | 0 | 0 | 1 |
| Post-resection | | 4[c] (2) | 0 | 0 | 1 (1) | 0 | 5 |
| 10 months to 11 years p.o. | 23 | | | | | | 22 |
| Post-exploration[a] | | 0 | 0 | 0 | 0 | 1 | 1 |
| Post-resection | | | | | | | |
| Recurred[a] | | 3[d] | 0 | 0 | 0 | 0 | 3[d] |
| Possibly recurring | | 4[d] (2) | 0 | 0 | 0 | 0 | 4[d] |
| Clear | | 14 (4) | 0 | 0 | 1 (1) | 0 | 15 |
| Total tests | 65 | | | | | | |
| Total patients | | 43 | 2 | 4 | 4 | 10 | 63 |

[a] Tumour-bearers.

[b] Numbers of tests lacking white-cell count and/or differential count in parentheses.

[cc, dd] Indicates a case in common.

FIG. 1.—Peripheral-blood basophil count in normal individuals, in non-malignant chest patients, and in patients with primary bronchial carcinoma tested at diagnosis, 6 weeks after operation and 10 months to 11 years after operation. ○ No tumour (or believed to be tumour-free); ◐ bronchial carcinoma not excluded; ◑ recurrence suspected; ● primary bronchial carcinoma pretreatment, inoperable or recurrent.

### RESULTS

Thick smears made from the same sample gave consistent results. In counts ranging from 1 to 110/$\mu$l the mean standard deviation was $3{\cdot}7 \pm 0{\cdot}25$ for 115 samples. In 18 tests on 7 normal individuals counts ranged from 9 to 29 with a median of 19/$\mu$l (Fig. 1).

The median values from samples from lung-cancer patients clinically free of disease after resection (16/$\mu$l all, 20/$\mu$l squamous $>10$ months p.o.) and from patients without malignant disease (13/$\mu$l) were similar, though the spread was wider in the latter group (Fig. 1); the highest value being shown by the only patient suffering from bronchial asthma (48/$\mu$l). Bronchial carcinoma-bearing patients (solid symbols, Fig. 1) tended to show higher values (median 33, range 5·5–110) significantly higher than the normal individuals ($P < 0{\cdot}002$), other patients ($P < 0{\cdot}001$) and the resected patients free of disease ($P < 0{\cdot}001$). In tumour bearers, values were higher than the highest normal value in 15/23 with squamous carcinoma, 2/8 with other types, and 7/10 cases of lung cancer without histopathological confirmation.

Of the 6 patients tested 6–8 weeks after operation, normal values were recorded for each of the 5 who had had resections but not for the patient who had undergone exploratory thoracotomy (Fig. 1). Counts tended to be high in patients with recurrent disease (Fig. 1) and in patients *bearing* tumours of other types (renal carcinoma 31/$\mu$l, resected oesophageal carcinoma 18/$\mu$l, non-Hodgkin's lymphoma 55·3/$\mu$l.)

Two patients with squamous carcinoma were each tested twice. In one a basophil count of $30{\cdot}3 \pm 5{\cdot}0$/$\mu$l on diagnosis fell to $12{\cdot}3 \pm 4{\cdot}6$/$\mu$l 6 weeks after resection; in the other a value of $17 \pm 4{\cdot}0$/$\mu$l 5 years after resection, when recurrence was suspected, rose to $33{\cdot}0 \pm 0{\cdot}7$/$\mu$l 4 months later, when recurrence was explicit.

In general, basophil counts were not related to total leucocyte counts, though significant correlation was seen in inoperable cancer patients without histological confirmation ($n = 9$, $r = 0{\cdot}68$, $P < 0{\cdot}05$) and in other chest patients with WBC counts under $10 \times 10^9$/l ($r = 0{\cdot}59$, $P < 0{\cdot}01$). There was a tendency throughout for the numbers of basophils and monocytes to correlate, significant only in tumour-bearers (Table II) and in other chest patients without leucocytosis ($r = 0{\cdot}53$, $P < 0{\cdot}01$). A suggestion of correlation between basophils and eosinophils was noted in bronchial carcinoma-bearing patients and in resected patients free of disease (Fig. 2) but not in the other groups.

TABLE II.—*Median values for leucocyte, lymphocyte, monocyte and eosinophil counts/μl and correlations with basophil counts in patients with primary bronchial carcinoma*

| | | Leucocytes × $10^{-3}$ | | Lymphocytes | | Monocytes | | Eosinophils | | Basophils | |
|---|---|---|---|---|---|---|---|---|---|---|---|
| | *n* | Median | R[a] | Median | R | Median | R | Median | R | *n* | Median |
| *All types* | | | | | | | | | | | |
| Tumour-bearers[b] | 36 | 7·4 | 0·48*[c] | 1449 | 0·23 | 665 | 0·41** | 135 | 0·35** | 41 | 33 |
| Inop. at diagnosis | 14 | 7·9 | | 1260 | | 589 | | 189 | | 18 | 28 |
| Later resected | 18 | 7·3 | | 1728**[d] | | 810 | | 129 | | 18 | 31 |
| *Squamous Ca.* | | | | | | | | | | | |
| Tumour-bearers | 20 | 7·3 | 0·22 | 1420 | 0·29 | 662 | 0·37 | 116 | 0·35 | 23 | 33 |
| Inop. at diagnosis | 6 | 7·0 | | 993 | | 573 | | 42 | | 9 | 23 |
| Later resected | 10 | 7·2 | | 1589*[d] | | 767 | | 135 | | 10 | 37·5**[d] |
| Post-resection (> 10 months), clear | 10 | 7·3 | −0·18 | 1776*[d] | 0·04 | 447 | 0·24 | 142 | 0·36 | 14 | 20 |

* $P < 0·01$, ** $P < 0·05$.
[a] Spearman's rank correlation coefficient for correlation with the basophil count.
[b] Includes patients post-exploration and recurred after resection (see Table I).
[c] Reduced (0·32, non-sig.) when patients with a high white-cell count ($\geqslant 10 \times 10^9$/l) excluded.
[d] For comparison with "Inop. at diagnosis" by Mann–Whitney *U*-test.

FIG. 2.—Peripheral-blood basophils and eosinophils in bronchial carcinoma patients bearing tumour ($r = 0·35$, $P < 0·05$, solid symbols) and clinically free of recurrence after resection ($r = 0·36$, non-sig. open symbols). A, on diagnosis of squamous carcinoma: ▲, subsequently resected; ●, inoperable. B, On diagnosis: ●, inoperable, histopathology not known; ▲, ■, ▼, adenocarcinoma, oat-cell carcinoma, large-cell undifferentiated carcinoma (respectively) subsequently resected. C, Squamous-cell-carcinoma patients tested during follow-up: △, resected, clinically clear; ▲, resected, recurring; ▼, not resected.

The highest basophil counts were seen in tumour-bearing patients with squamous bronchial carcinoma (Fig. 2A, C). Counts were not related to the differentiation of the tumour. Pretreatment samples from patients with subsequently resected squamous carcinomas tended to have higher counts of basophil, lymphocytes and monocytes than from those who were inoperable (Table II). If basophil counts were plotted against lymphocyte counts (Fig. 3) an arbitrary line could be drawn giving almost complete separation between the inoperable patients (below and

FIG. 3. Peripheral-blood basophils, lymphocytes and monocytes in pre-treatment samples from patients with squamous-cell bronchial carcinoma. ▲, Subsequently resected; ●, Inoperable.

TABLE III.—*Results of tests in 10 patients with non-squamous primary bronchial carcinoma*

| | Estimates of cells/µl: Lymphocyte | Monocyte | Eosinophil | Basophil count/µl | Tumour bearing | Tested |
|---|---|---|---|---|---|---|
| 1. Adenoca | 1739 | 884 | 211 | 27·8±1·8 | Yes | On diagnosis, later resected |
| 2. Adenoca m/d | 1790 | 1252 | 93 | 47·5±5·7 | Yes | On diagnosis, later resected |
| 3. Adenoca p/d | 735 | 200 | 121 | 6·5±3·5 | Yes | On diagnosis, later resected |
| 4. Adenoca | 1903 | 774 | 22 | 23·0±2·8 | Yes | On diagnosis, later resected |
| 5. Large-cell undifferentiated ca | 2166 | 358 | 122 | 13·0±2·8 | Yes | On diagnosis, later resected |
| 6. Large-cell undifferentiated ca | 132 | 362 | 28 | 15·3±5·3 | Yes | On diagnosis, later resected |
| 7. Small (oat-) cell ca | 2945 | 1053 | 389 | 49·5±4·2 | Yes | On diagnosis, later resected |
| 8. Small (oat-) cell ca | 2198 | 1217 | 141 | 5·5±0 | Yes | On diagnosis, later resected |
| 9. Large-cell undifferentiated ca | ND | ND | ND | 14±3 | No | 6 weeks post-resection |
| 10. Large-cell undifferentiated ca | ND | ND | ND | 19±4 | No | 10 years post-resection |

to the left) and those subsequently resected (above and to the right). When the same line was applied to the other plots, 17/18 normal samples and 25/29 from other chest patients fell below and to the left of the line: the exceptions included 2 patients in whom the diagnosis of bronchial carcinoma has not been finally excluded, and 1 with bronchial asthma. The pattern of basophil/monocyte plots was similar; an arbitrary line again separated most of the operable from the inoperable cases, but separation was less distinct and 9/27 other chest patients gave values above and to the right of the line including the same 3 patients and 6 others, all with infections. The pattern was less evident on basophil/eosinophil plots (Fig. 2A).

Ten tests were performed in patients with other types of bronchial carcinoma (Table III, Fig. 2). Patients with adenocarcinomas and oat-cell carcinomas showed raised values but all 4 counts in patients with large-cell undifferentiated carcinomas (2 tumour bearers) were under 20/$\mu$l. The arbitrary dividing lines on basophil/lymphocyte/monocyte plots did not predict surgical resectability in these patients.

### DISCUSSION

This study has shown raised basophil counts in peripheral blood of most tumour-bearing patients with bronchial carcinoma, particularly those with squamous carcinoma. Higher counts were also noted in 3 patients in whom the diagnosis of bronchial carcinoma has not been finally excluded, but in only 2 patients from the same wards without malignant disease, one of whom was the only case of bronchial asthma.

Only one other group has reported studies of blood basophils in cancer patients (Gracheva *et al.*, 1976; Sergeev *et al.*, 1977) and they found increased degranulation but no consistent differences in the numbers of blood basophils in patients with carcinoma of the stomach. Degranulation was particularly marked in patients with more advanced disease (Sergeev *et al.*, 1977). The few patients who showed positive responses (6/56) in delayed hypersensitivity reaction skin tests with a membrane-antigen preparation from stomach carcinoma tended to have higher basophil counts and more degranulation of basophils than patients who did not respond. Controls in their study gave higher values ($35{\cdot}1 \pm 2{\cdot}7/\mu$l) than in ours (median 19/$\mu$l, mean $19{\cdot}9 \pm 7{\cdot}0$) consistent with the differences reported by Hirsch *et al.* (1974) for the chamber method (which they used) and the thick-smear method. Controls in our study gave values very close to those of Hirsch & Kalbfleich (1976) for non-atopic individuals ($17{\cdot}9 \pm 1{\cdot}2$ winter, $20{\cdot}8 \pm 1{\cdot}3$ summer), using the thick-smear method.

The only well-documented reports of increased circulating basophils in man (except in the leukaemias) are in patients recovering from infection (Mitchell, 1958), with ulcerative colitis (Juhlin, 1963), and those with severe atopic disease, all conditions with immunological components. In this study increased basophils in squamous-carcinoma patients were not associated with a raised leucocyte count, nor were basophil counts altered in most of the other patients with chronic bronchitis, tuberculosis and other infections.

Tissue infiltration with basophils occurs in the relatively transient cutaneous basophil hypersensitivity, but less in classical delayed hypersensitivity, apparently because of an inhibitory effect (Dvorak *et al.*, 1980). Basophils were noted in close contact with dying cells of the Line 1 hepatoma in immunized guinea-pigs (Dvorak *et al.*, 1973) and were prominent in the cellular infiltrate in the skin-window technique when autologous breast-tumour sections were applied in strongly reactive patients (Black & Leis, 1971). Eosinophil (Kolb & Muller, 1979), mononuclear (Ioachim *et al.*, 1976) and macrophage (Kolb & Muller, 1979) infiltration of lung carcinomas were each reported to be associated with a better prognosis, but the significance of mast cells and basophils has not been investigated. Blood basophilia was noted in guinea-pigs rejecting a transplanted hepatoma (Dvorak *et al.*, 1973).

With some techniques, cell-mediated tumour immunity may be detected *in vitro* from early on in tumour development to many years after resection (Halliday, 1977). Antigen, continuously shed from tumour cells, was detectable in the serum of tumour bearers, but the concentration fell rapidly after tumour excision (Baldwin *et al.*, 1973). The raised basophil counts in patients with active lung cancer probably reflect naturally occurring challenge of an anti-tumour immune response by circulating antigen. This interpretation is supported by the normal counts in patients 6 weeks after resection

(but not after exploration) and in resected patients free of recurrence.

Lymphocytosis occurred in mice during rejection, and *induced* lymphocytosis was the earliest effective anti-tumour adjuvant therapy (Murphy, 1926). Reactive lymphocytosis (Anthony *et al.*, 1975) and increased lymphocyte mitogenesis (Chretien *et al.*, 1973) have been reported in some lung-cancer patients, returning to normal levels after lung resection.

The correlation noted between a function of the lymphocyte and basophil counts and resectability may be evidence that the resistance mechanisms they reflect are exerting some control over tumour extension as indicated for lymphocytes in a previous study (Anthony *et al.*, 1981). However, it could also result from earlier diagnosis in patients with chronic basophil release due, for instance, to exacerbation of the presenting dyspnoea by a degree of bronchospasm due to histamine (Bhat *et al.*, 1976).

The numbers of basophils in the blood of lung-cancer patients showed some correlation with the numbers of monocytes, and of eosinophils, stronger and more consistent in each case than that with the overall leucocyte count, on which they would be unlikely to depend. The correlation between basophils and eosinophils was not seen in normal individuals or in the diverse group of control patients, though it could have been missed because of the low counts with their relatively large errors of enumeration. The numbers of basophils and eosinophils correlated similarly in lung-cancer patients bearing tumours and in those believed to be free of disease after resection, significant in the former group. The correlation could result from a common homoeostatic mechanism controlling levels of both types of cell, or from influences of one cell type on the circulating levels of the other. Little is known about the control of basophils, or about their lifespan, since the only evidence has been from studies of abnormal basophils. Basophil mediators are reported to attract eosinophils to the site of nematode infestation and arm them for more potent killing of the nematodes (Kay, 1980). It is possible that the link between the numbers of circulating basophils and eosinophils in lung cancer patients lies in a similar interaction in relation to the tumour, since eosinophil infiltration of tumours was associated with both blood eosinophilia and with a better prognosis (Kolb & Muller, 1979).

Using the thick-smear method for the blood basophil count, which is a relatively simple test, we found raised basophil counts in most patients with resectable squamous-cell carcinomas and in some patients with carcinomas of other types. The data suggest that a normal basophil count is uninformative but that a raised basophil count in patients without major allergy should be another factor to consider in the differential diagnosis of carcinoma of the bronchus and possibly with carcinomas at other sites.

I am indebted to Mrs L. Bloomer and Miss B. Andrew for technical assistance, Dr T. Mueller and Dr K. Madsen for their help, Mr D. A. Watson, Mr D. Walker, Dr N. Cooke and Dr M. Muers for access to their patients and the Yorkshire Cancer Research Campaign for financial support.

### REFERENCES

Anthony, H. M., Kirk, J. A., Madsen, K. E., Mason, M. K. & Templeman, G. H. (1975) E and EAC rosetting lymphocytes in patients with carcinoma of bronchus. II. A sequential study of thirty patients: effect of BCG. *Clin. Exp. Immunol.*, **20**, 41.

Anthony, H. M., Madsen, K. E., Mason, M. K. & Templeman, G. H. (1981) Lung cancer—immune status, histopathology and smoking. Is oat cell carcinoma lymphodependent? *Br. J. Dis. Chest.*, **75**, 40.

Askenase, P. W. (1979) Mechanisms of hypersensitivity: Cellular interactions, basophil arrival and function in tissue hypersensitivity reactions. *J. Allergy Clin. Immunol.*, **64**, 79.

Baldwin, R. W., Embleton, M. J. & Robins, R. A. (1973) Cellular and humoral immune reactions to rat hepatoma-specific antigens correlated with tumour status. *Int. J. Cancer*, **11**, 1.

Bhat, K. N., Arroyave, C. M., Marney, S. R., Stevenson, D. D. & Tan, E. M. (1976) Plasma histamine changes during provoked bronchospasm in asthmatic patients. *J. Allergy Clin. Immunol.*, **58**, 647.

Black, M. & Leis, H. P. Cellular responses to autologous breast cancer tissue. *Cancer*, **28**, 263.

Charles, T. J., Williams, S. J., Seaton, A., Bruce, C. & Taylor, W. H. (1979) Histamine basophils and eosinophils in severe asthma. *Clin. Sci.*, **57**, 39.

CHRETIEN, P. B., CROWDER, W. L., GERTNER, H. R., SAMPLE, W. F. & CATALONA, W. J. (1973) Correlation of pre-operative lymphocyte reactivity with the clinical course of cancer patients. *Sur. Gynaecol. Obstet.*, **136**, 380.

CHURCH, M. K. & HOLGATE, S. T. (1980) The basophil leucocyte: Morphological, immunological and biochemical considerations. In *Topical Reviews in Haematology* (Ed. Roath). Bristol: John Wright. p. 65.

DVORAK, H. F., DVORAK, A. M. & CHURCHILL, W. H. (1973) Immunologic rejection of diethylstilboestrol-induced hepatomas in Strain-2 guinea-pigs, Participation of basophilic leukocytes and macrophage aggregates. *J. Exp. Med.*, **137**, 751.

DVORAK, H. F., GALLI, S. J. & DVORAK, A. M. (1980) Expression of Cell-Mediated Hypersensitivity *in vivo*—recent advances. *Int. Rev. Exp. Pathol.*, **21**, 120.

EASTHAM, R. D. (1977) *Clinical Haematology, 5th edn.* Bristol: John Wright. p. 148.

GRACHEVA, Z. A., BABAKOVA, S. V., GORODILOVA, V. V. & SOKOVA, I. I. (1976) The state of blood basophilic granulocytes in the estimation of sensibilisation of the organism of gastric cancer. patients. *Vopr. Onkol.*, **22**, 19.

HALLIDAY, W. J., MALUISH, A. E., STEPHENSON, P. M. & DAVIS, N. S. (1977) An evaluation of leukocyte adherence inhibition in the immunodiagnosis of colorectal cancer. *Cancer Res.*, **37**, 1971.

HIRSCH, S. R. & KALBFLEICH, J. H (1976) Circulating basophils in normal subjects and subjects with hay fever. *J. Allergy Clin. Immunol.*, **58**, 676.

HIRSCH, S. R., RIMM, A. A. & ZASTROW, J. E. (1974) The absolute peripheral basophil count. *J. Allergy Clin. Immunol.*, **53**, 303.

IOACHIM, H. L., DORSETT, B. H. & PALUCH, E. (1976) The immune response at the tumour site in lung carcinoma. *Cancer*, **38**, 2296.

JUHLIN, L. (1963) Basophil leucocytes in ulcerative colitis. *Acta Med. Scand.*, **176**, 351.

KAY, A. B. (1980) The role of the eosinophil in physiological and pathological processes. In *Recent Advances in Clin. Immunology* **2**, (Ed. Thompson), Edinburgh: Churchill Livingstone.

KIMURA, I., MORITANI, Y. & TANIZAKI, Y. (1973) Basophils in bronchial asthma with reference to reagin-type allergy. *Clin. Allergy*, **3**, 195.

KOLB, E. & MULLER, E. (1979) Local responses in primary and secondary human lung cancer. II Clinical considerations. *Br. J. Cancer*, **40**, 410.

MITCHELL, R. G. (1958) Basophilic leucocytes in children in health and disease. *Arch. Dis. Childh.*, **33**, 193.

MOORE, J. E. & JAMES, G. W. (1953) Simple direct method for Absolute Basophil Leucocyte Count. *Proc. Soc. Exp. Biol. Med.*, **82**, 601.

MURPHY, J. B. (1926) The lymphocyte in resistance to tissue grafting, malignant disease and tuberculous infection: An experimental study. *Rockefeller Inst. Med. Res. Monog.*, **21**.

ORFANAKIS, N. G., OSTLUND, R. E., BISHOP, C. R. & ATHENS, J. W. (1970) Normal blood leucocyte concentration values. *Am. J. Clin. Pathol.*, **53**, 647.

SAAVEDRA-DELGADO, A. M., MATHEWS, K. P., PAN, P. M., KAY, D. R. & MUILENBERG, M. L. (1980) Dose response studies of the suppression of whole blood histamine and basophil counts by prednisone. *J. Allergy Clin. Immunol.*, **66**, 464.

SERGEEV, S. I., GRACHEVA, Z. A., BERGUT, F. A. & SOKOVA, I. I. (1977) Degranulation and intracellular heparin of basophilic granulocytes in the blood of patients with carcinoma of the stomach. *Sov. Med.*, **2**, 29.

WINTROBE, M. M. (1974) *Clinical Haematology, 7th edn.* Philadelphia: Lea & Febiger, p. 1286.